# Supplementary material for: Dynamic transcriptomic profiles of zebrafish gills in response to zinc supplementation
Source: BMC Genomics. 2010 Oct 11;11:553. doi: 10.1186/1471-2164-11-553 (PMC3091702; doi:10.1186/1471-2164-11-553)
Supplement: Additional file 2 — Interactive Direct Interaction Network representing the molecular interactions between zinc, copper, iron, calcium and proteins encoded by transcripts changed by zinc supplementation. Mini web-site containing index.html and hyperlinked pages in subdirectory describing a Direct Interaction Network automatically generated based on curated interactions contained within the proprietary PathwayArchitect database. Ovals represent proteins and the circles symbolize metal ions. Objects are coloured by their abundance in zebrafish at the time-point they were significantly different from the control is a scale from -4 fold (dark green) to +4 fold (dark red). Where significant differences were found at more than one time-point, the colour overlay shows expression at the first instance. Dark blue squares denote 'binding', and light blue squares 'expression'; green squares stand for 'regulation', green diamonds for 'metabolism', and green circles for 'promoter binding'. Arrow heads indicate directionality of the interaction where annotated. All nodes and edges can be further interrogated by selecting the relative area of the image. [file 1471-2164-11-553-S2.zip › PathwayArchitect Zn xs DIN/95552.html]

# PROTEIN: CDC25A

|  |  |
| --- | --- |
| Name | CDC25A |
| Type | PROTEIN |
| Description | cell division cycle 25A |
| Note | CDC25A is a member of the CDC25 family of phosphatases. CDC25A is required for progression from G1 to the S phase of the cell cycle. It activates the cyclin-dependent kinase CDC2 by removing two phosphate groups. CDC25A is specifically degraded in response to DNA damage, which prevents cells with chromosomal abnormalities from progressing through cell division. CDC25A is an oncogene, although its exact role in oncogenesis has not been demonstrated. Two transcript variants encoding different isoforms have been found for this gene. |
| Alias | Cdc25a |
|  | Dual specificity phosphatase Cdc25A |
|  | D9Ertd393e |
|  | cell division cycle 25 homolog A |
|  | cell division cycle 25A |
|  | dual specificity phosphatase CDC25A |
|  | CDC25A2 |
|  | CDC25A |
|  | Cdc25m3 |
|  | M-phase inducer phosphatase 1 |


---

|  |  |
| --- | --- |
| GO Component | cellular component unknown |
|  | intracellular |


---

|  |  |
| --- | --- |
| GO ID | GO:0000087 |
|  | GO:0005622 |
|  | GO:0000910 |
|  | GO:0004725 |
|  | GO:0007067 |
|  | GO:0008372 |
|  | GO:0016787 |
|  | GO:0008283 |
|  | GO:0004721 |
|  | GO:0051301 |
|  | GO:0000082 |
|  | GO:0004727 |
|  | GO:0006470 |
|  | GO:0007049 |
|  | GO:0000079 |


---

|  |  |
| --- | --- |
| MIM | MIM:116947 |


---

|  |  |
| --- | --- |
| Connectivity | 291 |


---

|  |  |
| --- | --- |
| Entrez ID | 993 |
|  | 171102 |
|  | 12530 |


---

|  |  |
| --- | --- |
| Agilent ID | A\_14\_P113602 |
|  | A\_52\_P282729 |
|  | A\_53\_P149270 |
|  | A\_42\_P834104 |
|  | A\_51\_P519319 |
|  | A\_24\_P397107 |
|  | A\_14\_P129247 |
|  | A\_53\_P146642 |
|  | A\_23\_P121423 |
|  | A\_44\_P362579 |
|  | A\_14\_P129651 |


---

|  |  |
| --- | --- |
| Cellular Localization | Cell |


---

|  |  |
| --- | --- |
| DbXref | KEGG pathway##04110##Cell cycle##http://www.genome.jp/dbget-bin/show\_pathway?hsa04110+993 |
|  | Reactome##172757##Cell Cycle Checkpoints##http://www.reactome.org/cgi-bin/eventbrowser?DB=gk\_current&ID=172757 |
|  | KEGG pathway##04110##Cell cycle##http://www.genome.jp/dbget-bin/show\_pathway?mmu04110+12530 |
|  | Reactome##172742##Cell Cycle (Mitotic)##http://www.reactome.org/cgi-bin/eventbrowser?DB=gk\_current&ID=172742 |
|  | Reactome##69278##Cell Cycle (Mitotic)##http://www.reactome.org/cgi-bin/eventbrowser?DB=gk\_current&ID=69278 |
|  | Reactome##69620##Cell Cycle Checkpoints##http://www.reactome.org/cgi-bin/eventbrowser?DB=gk\_current&ID=69620 |
|  | KEGG pathway##04110##Cell cycle##http://www.genome.jp/dbget-bin/show\_pathway?rno04110+171102 |


---

|  |  |
| --- | --- |
| Pathway | P53 Signaling |
|  | Zn xs inventory |
|  | Zn xs DIN |


---

|  |  |
| --- | --- |
| GO Process | cell proliferation |
|  | mitosis |
|  | protein amino acid dephosphorylation |
|  | regulation of cyclin dependent protein kinase activity |
|  | M phase of mitotic cell cycle |
|  | cell cycle |
|  | G1/S transition of mitotic cell cycle |
|  | cell division |
|  | cytokinesis |


---

|  |  |
| --- | --- |
| UniGene | Rn.11390 |
|  | Hs.1634 |
|  | Mm.307103 |


---

|  |  |
| --- | --- |
| Affymetrix Probeset ID | 135624\_f\_at |
|  | 1375683\_at |
|  | 1377558\_at |
|  | 1387371\_at |
|  | 1417131\_at |
|  | 1417132\_at |
|  | 1555772\_a\_at |
|  | 1738\_at |
|  | 204695\_at |
|  | 204696\_s\_at |
|  | 35407\_at |
|  | 54538\_at |
|  | 97389\_at |
|  | 97390\_at |
|  | aa272465\_s\_at |
|  | g4502704\_3p\_a\_at |
|  | Hs.1634.0.S2\_3p\_at |
|  | Hs2.1634.2.S1\_3p\_s\_at |
|  | M81933\_at |
|  | rc\_AI059933\_at |
|  | U27323\_s\_at |
|  | 1445995\_at |
|  | RC\_AA071514\_at |
|  | TC23221\_at |
|  | TC23644\_at |
|  | TC34653\_at |


---

|  |  |
| --- | --- |
| EC Number | EC 3.1.3.48 |


---

|  |  |
| --- | --- |
| GO Function | hydrolase activity |
|  | prenylated protein tyrosine phosphatase activity |
|  | protein tyrosine phosphatase activity |
|  | phosphoprotein phosphatase activity |


---

|  |  |
| --- | --- |
| Nucleotide | BQ065803 |
|  | C85103 |
|  | NM\_133571 |
|  | BX392016 |
|  | AF277722 |
|  | AY137580 |
|  | AK077609 |
|  | BC007401 |
|  | NM\_007658 |
|  | BC046296 |
|  | AI917350 |
|  | BC018642 |
|  | D16236 |
|  | NM\_201567 |
|  | AK077991 |
|  | NM\_001789 |
|  | AF527417 |
|  | M81933 |
|  | U27323 |
|  | AK142334 |
|  | AF112978 |
|  | AK036499 |
|  | AJ242714 |


---

|  |  |
| --- | --- |
| Protein | BAE25035 |
|  | P48965 |
|  | NP\_031684 |
|  | AAH46296 |
|  | AAH18642 |
|  | AAM77917 |
|  | P30304 |
|  | NP\_598255 |
|  | NP\_963861 |
|  | AAN11305 |
|  | AAA58415 |
|  | P48964 |
|  | BAA03761 |
|  | AAG41884 |
|  | AAH07401 |
|  | AAA85580 |
|  | NP\_001780 |


---

|  |  |
| --- | --- |
| Organism | Mammal |


---

|  |  |
| --- | --- |
| Location | chromosome 3, 3p21 (Homo sapiens) |
|  | 9 62.0 cM (Mus musculus) |
|  | chromosome 9, 9 62.0 cM, 9 F2 (Mus musculus) |
|  | chromosome 8, 8q32 (Rattus norvegicus) |


---

|  |  |
| --- | --- |
